# Factors affecting medium-term patient satisfaction after arthroscopic repair of small to medium-sized rotator cuff tears: An observational study

**DOI:** 10.1097/MD.0000000000038211

**Published:** 2024-05-17

**Authors:** Onur Hapa, Selahaddin Aydemir, Emre Acar, Ali Cantürk, Berkay Yanik, Gürhan Tükel, Onur Gürsan, Ali Balci

**Affiliations:** a Department of Orthopaedics and Traumatology, Dokuz Eylül University Hospital, İzmir, Turkey; b Department of Radiology, Dokuz Eylül University Hospital, İzmir, Turkey; c Department of Orthopaedics and Traumatology, Urla State Hospital, İzmir, Turkey.

**Keywords:** rotator cuff, shoulder arthroscopy, patient satisfaction

## Abstract

This study aimed to assess the effect of the status of the tendon and patient factors on patient satisfaction after rotator cuff repair. Forty-six patients treated for tears with a minimum of 5-year follow-up were included. Gender, age, and active smoking status were recorded. Pain visual analogue scale, American Shoulder and Elbow Surgeons Standardized Shoulder Assessment Form, Simple Shoulder Test, and Single Assessment Numeric Evaluation were recorded preoperatively and at the last follow-up. Patients were divided into groups of highly satisfied (HS) and vaguely satisfied (VS) patients. Patients were evaluated with MRI both preoperatively and at their last follow-up. Of the 46 patients, 17 were HS and 29 were VS. The HS group had 7 re-ruptures, 4 of which were progressed tears, whereas the VS group had 15 re-ruptures, 4 of which were progressed tears. There was no difference in the rate of re-ruptures or progressed tears between groups. The HS group had a higher frequency of males. However, frequencies of active smoking or osteoarthritis of grade 2 or higher were lower in the HS group. It was shown that patient satisfaction after repair depends on patient-related factors like gender and smoking rather than tendon healing or degeneration.

## 1. Introduction

Arthroscopic rotator cuff (RC) tear repair is now considered the gold standard for the treatment of most RC tears, providing similar functional results to open and mini-open surgery, with lesser morbidity.^[1]^ The patient-acceptable symptom state (PASS) has emerged as a metric for evaluating patient satisfaction after treatment. There is little research on factors affecting PASS values after arthroscopic RC repair. The few studies only reported 1-year PASS values for shoulder scoring systems and these studies reported a range of different values.^[2–6]^

Another controversial issue is whether healing of the tendon after repair affects patients’ functional scores and/or satisfaction.

The most recent meta-analysis reported that there may be better functional scores in patients with healed tendons compared to non-healed patients. However, the results did not demonstrate clinical significance and the study mentioned limitations such as substantial heterogeneity, inconsistent definitions of retear, and few high-quality studies with longer-term follow-ups.^[7]^ Reported results were worse in patients with tears that progressed to a greater size compared to their preoperative size.^[6,8]^

This study aimed to assess the effect of the healing status and integrity of the tendon at the last follow-up, and to clarify the effect of various variables on patient satisfaction at a minimum of 5 years after arthroscopic RC repair. The hypothesis was that tears that had progressed (tear size similar or increased compared to the preoperative MRI) at the last follow-up would be correlated to a lesser degree of satisfaction.

## 2. Methods

The study was approved by the Ethical Committee for Clinical Research, and informed consent was obtained from all patients.

**Figure 1.**
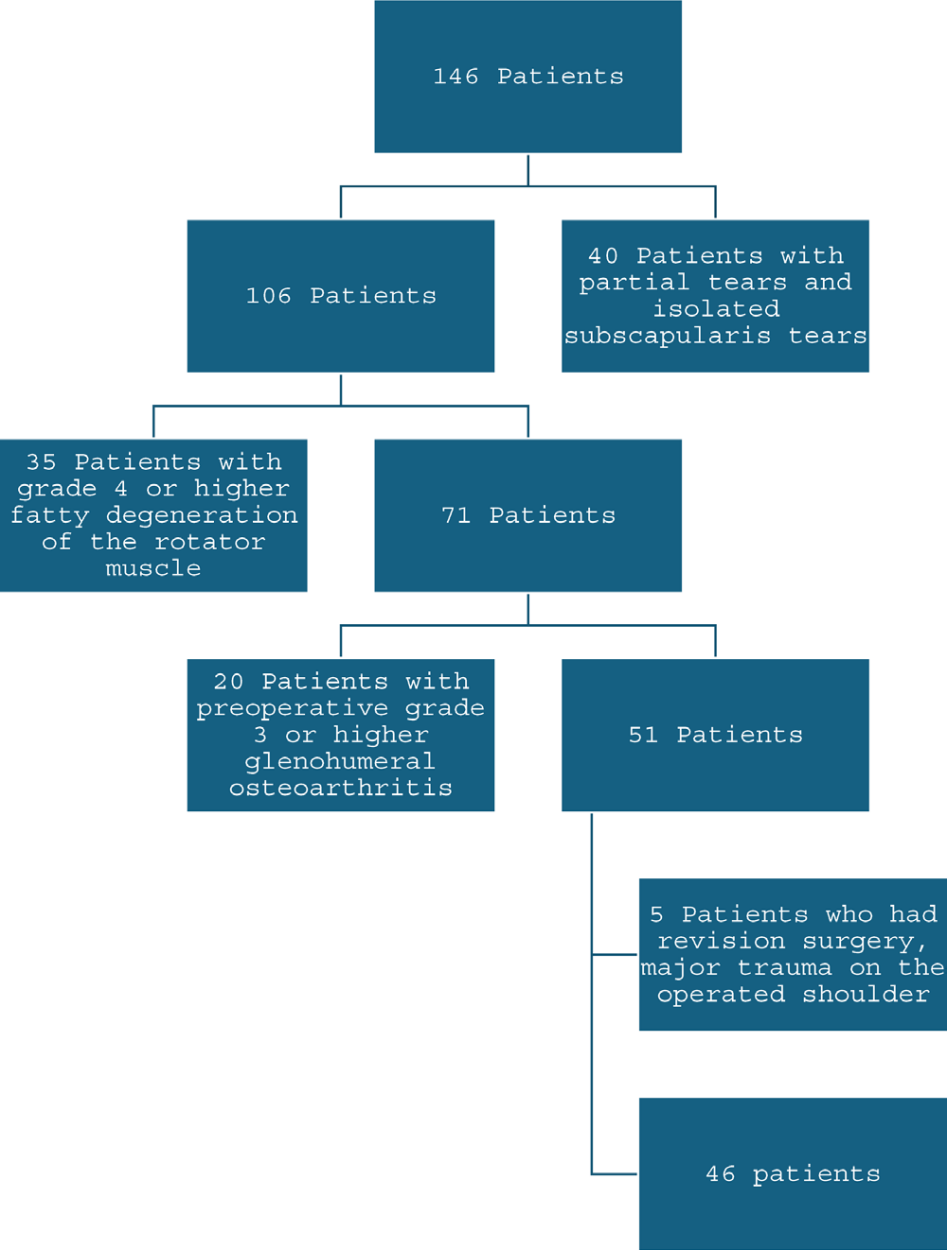
Flowchart.

In this single-center study patients who were diagnosed with RC tears and had arthroscopic RC repair performed between 2013 and 2018 with a minimum of 5 years of follow-ups were assessed. Surgery was performed, by a single surgeon, on patients with full-thickness tears that did not show improvement after 3 months of physical therapy. Patients with partial tears, isolated subscapularis tears, grade 4 or higher fatty degeneration of the rotator muscle, patients who had revision surgery, major trauma on the operated shoulder, and preoperative grade 3 or higher glenohumeral osteoarthritis (OA) were excluded from the study. After applying the exclusion criteria, 46 patients (29 female, 17 male) were included in this study (Fig. 1). The mean age of the patients was 55 ± 9 years and the mean follow-up time was 84 ± 14 months (Table 1).

**Table 1:** Patient demographic data.

	HS	VS
Age (yr) (mean ± standard deviation)	53 ± 11	55 ± 8
Gender: male/female	10/7	8/21
Follow-up (mo) (mean ± standard deviation)	83 ± 12	84 ± 15
Smoking patients	1	9
Diabetes positive	6	8
Education level of patients college/high school	6/11	5/24
Subscapularis repair	2	4
Biceps tenotomy	4	11
Tear size small/medium/large	12/4/1	14/13/2
Preop OA grade I/II	16/1	21/8
Post op OA grade I/II/IV	16/1	21/7/1
Preop degen grade 0/1/2	14/2/1	19/7/3
Postop degen grade 0/1/2	10/7	12/11/6

HS = highly satisfied, OA = osteoarthritis (Samilson and Prieto Classification), VS = vaguely satisfied.

Demographic data such as gender, age, medical history (diabetes mellitus), education level, and active smoking status were recorded. In addition, imaging and operative data such as tear size according to the Cofield classification (small, medium, large, or massive),^[9]^ modified Goutallier fatty infiltration,^[10,11]^ the number of anchors used for the repair, biceps procedure (none, tenotomy), and subscapularis tear and repair were also collected.

Patients’ MRIs taken preoperatively and at their last follow-up were evaluated separately by 2 radiologists. The Sugaya classification was used to assess tendon healing and integrity, and Types IV and V were considered retears.^[12]^

Retears confirmed on MRI were reevaluated to determine the retear size and dichotomized the patients into 2 groups consisting of those with retears smaller than the preoperative tear size and those with retears of the same size or larger than the preoperative tear size. The latter was defined as a “progressed retears.”^[6]^ The presence of shoulder OA was classified using shoulder X-rays according to the classification of Samilson and Prieto.^[13]^

The patients’ pain visual analogue scale (PVAS), American Shoulder and Elbow Surgeons Standardized Shoulder Assessment Form (ASES),^[14]^ Simple Shoulder Test (SST),^[15]^ and Single Assessment Numeric Evaluation (SANE)^[16]^ scores were assessed preoperatively and at their final follow-up. Anchor questions were used to define the PASS values for PVAS, ASES, SST, and SANE.^[17,18]^

Patients were divided into 2 main groups consisting of those who were highly satisfied (HS) and vaguely satisfied (VS). Patients were asked about their satisfaction levels and were instructed to choose an answer that represented their current situation (Table 2). Highly satisfied patients were considered as those who stated that their shoulders were completely healed. Vaguely satisfied patients consisted of: satisfied patients who had symptoms with activity, somewhat satisfied patients who had minor symptoms, dissatisfied patients whose symptoms were the same as before surgery, and severely dissatisfied patients who were worse off after surgery.

**Table 2:** Grouping of patient satisfaction levels.

Very satisfied	My shoulder has healed completely
Satisfied	I have only minor, activity-related symptoms. My shoulder is much better than before treatment
Somewhat satisfied	I have only minor symptoms. My shoulder is better than before treatment
Dissatisfied	My shoulder is the same as before treatment
Very dissatisfied	My shoulder is worse than before treatment

For the univariable analysis of the predictors of patient dissatisfaction, Fisher exact tests were used to compare categorical variables, and variables with *P* < .20 were included in the multivariate analysis. For nonparametric data, a Mann–Whitney test was performed.

Nonparametric receiver operating characteristic (ROC) curves and area under the ROC curve (AUC) analysis were used to evaluate each outcome score to predict PASS, based on the above anchor method calculation. An AUC > 0.8 was considered as excellent.^[19]^ For the ROC curve analysis, the optimum cutoff value between the VS and HS groups was determined to be the PASS value (sensitivity-based and specificity-based approach).^[9]^ *P* < .05 was considered to be statistically significant. Statistical analyses were performed using IBM SPSS, version 21.0.0.0, for Windows (IBM Corp, Armonk, NY, USA).

### 2.1. Surgical technique

The patient was positioned in the lateral decubitus position. After diagnostic arthroscopy, a tenotomy of the biceps was performed if an associated lesion of the long head of the biceps tendon was observed. If a subscapularis tendon tear was seen to be present, it was repaired to the lesser tuberosity. Depending on the tear size, the supraspinatus tendon was repaired using the single-row or suture-bridge technique. In the instance of a severe acromial spur, acromioplasty was performed.

## 3. Results

There were 17 HS patients and 29 VS patients.

Of the 29 VS patients, 22 were satisfied, 6 were somewhat satisfied and 1 patient was dissatisfied, with symptoms the same as before their surgery. HS consisted of 7 female and 10 male patients with 83 ± 12 months of follow-up, and VS group had 8 female and 21 male patients with 84 ± 15 months of follow-up. The mean ASES, SANE, SST, and PVAS scores at the last follow-ups were significantly better than preoperative values in both groups (HS group ASES, SANE, SST, PVAS *P* = .000, *P* = .000, *P* = .000, *P* = .000, VS group ASES, SANE, SST, PVAS *P* = .000, *P* = .000, *P* = .000, *P* = .000, respectively). However, scores at the last follow-ups were significantly better for ASES, SANE, SST, and PVAS in the HS group compared to the VS group (*P* = .000, *P* = .000, *P* = .000, *P* = .000, respectively).(Table 3).

**Table 3:** Functional scores of groups.

N: 46	ASES_pre_	ASES_po_	SANE_pre_	SANE_po_	SST_pre_	SST_po_	PVAS_pre_	PVAS_po_
HS	28 ± 14	95 ± 5	35 ± 14	97 ± 6	12 ± 13	90 ± 9	7 ± 1	0.2 ± 0.5
VS	20 ± 9	72 ± 15	27 ± 14	77 ± 14	10 ± 14	66 ± 16	8 ± 1	2 ± 1
*P* value	0.08	0.000	0.06	0.000	0.4	0.000	0.2	0.000

ASES = American Shoulder and Elbow Surgeons Standardized Shoulder Assessment Form, HS = highly satisfied, PVAS = pain visual analogue scale, SANE = Single Assessment Numeric Evaluation, SST = Simple Shoulder Test, VS = vaguely satisfied.

There were 22 re-ruptures (47,8%), 8 of which were classified as progressed retears. The HS group had 7 (41%) re-ruptures, 4 of which were progressed tears, whereas the VS group had 15 (51%) re-ruptures, 4 of which were progressed tears. There was no significant difference in the rate of frequency of re-ruptures or progressed tears between the HS and VS groups (p:0.4). At univariate analysis, smoking status, grade of OA, tear size, rates of re-rupture, fatty degeneration and progressed re-ruptures did not show a significant difference between HS and VS groups (*P* = .06, *P* = .07, *P* = .4, *P* = .02, *P* = .4, respectively). However, the frequency of male patients was higher in the HS group compared to the VS group (58% vs 27% p:0.03) (Table 4). At multivariate analysis, frequencies of active smoking and higher grades of OA were significantly lower in the HS group (*P* = .023, *P* = .027, respectively) (Table 4). The AUCs of ROC curves for all scores were >0.8 (i.e., excellent) (Table 5). The PASS was 0.5, 91.5, 92.5, and 79.1 for PVAS, ASES, SANE, and SST, respectively.

**Table 4:** Statistical analysis of variables.

Variable	Univariate analysis	Multivariate logistic regression analysis
Gender	0.03	0.017
Smoking	0.06	0.023
Osteoarthritis (grade 1 vs higher)	0.07	0.027
Tear size (small vs higher)	0.14	0.14
Re-rupture	0.4	
Fatty degeneration	0.2	0.2
Re-rupture progress	0.4	

**Table 5:** Cutoff values of scores.

Outcome measures	PASS value	Sensitivity	Specificity	AUC
PVAS	0.5	0.81	0.89	0.9
ASES	91.5	0.87	0.89	0.95
SANE	92.5	0.87	0.93	0.94
SST	79.1	0.87	0.72	0.9

ASES = American Shoulder and Elbow Surgeons Standardized Shoulder Assessment Form, AUC = area under the ROC curve, PASS = patient-acceptable symptom state, PVAS = pain visual analogue scale, SANE = Single Assessment Numeric Evaluation, SST = Simple Shoulder Test.

## 4. Discussion

The main finding of the present study was that men with a lesser degree of shoulder OA who do not smoke are very likely to be HS with arthroscopic RC repair at a minimum of a 5-year follow-up.

Most studies have reported postoperative satisfaction cutoff points of scores at 1 year; only the study by Kim et al^[4]^ has reported PASS values of 0.5, 93.5, and 82.5 for PVAS, ASES, and SANE scores at a minimum of 2 years of follow-ups.

One year postoperative PASS score was 1.7 for PVAS and scores varied between 78 and 86.7 for ASES and 71 and 82.5 for SANE.^[2,3,5,6]^ The different results in the present study were due to a longer period of follow-ups of at least 5 years and the distinction between HS versus VS instead of satisfied and unsatisfied as was dichotomized in former studies. However, we think our distinction based on a 5-level satisfaction system is more relevant in the case of medium-term follow-up. The above-mentioned studies have also reported separately female gender and smoking as factors for dissatisfaction similar to the present study.^[2,5,6]^

Another controversial issue is the effect of tendon re-rupture on the functional results of patients. The most recent meta-analysis reported that retears may have a negative impact on pain and function but judged this to be of minor clinical importance.^[7]^ Another study tried to explain this issue by suggesting that re-rupture needed to be bigger than the preoperative size of the original rupture to be clinically significant. It was reported that out of 117 patients 17 had re-ruptures, 9 of which were progressed tears. It was found that retear alone did not affect patient satisfaction. However, progressed retear size featured a significantly higher risk of patient dissatisfaction.^[6]^ The probable reason why the current study did not reach a similar conclusion was because the patient group had smaller and less degenerated tears. It was also proven in another study that tears with worse prognoses were larger in the anteroposterior plane and the tendons had a higher degree of fatty degeneration.^[8]^ The present study was probably unable to detect the effect of tear size and the effect of progressed tears on functional results because of the patient group, which mostly presented small and medium-sized tears.

The negative effects of factors such as smoking and female gender on outcomes have been reported in previous studies. Nicholson et al^[20]^ reported that smoking was the only factor significantly adversely influencing patient-reported outcomes. Malavolta et al^[21]^ did not find that smoking affected clinical results. This was probably due to the inclusion criteria used in their study for determining a patient as a smoker which grouped former smokers and active smokers together as opposed to only active smokers in the present study. However, they also cited male gender as being an independent prognostic factor for better results. Similar to the present study, Nabergoj et al^[22]^ also reported that female gender was a factor that negatively influenced the final clinical outcome and that healing of the tendon was not the sole factor for achieving satisfying outcomes. The tears were low-grade, as in our study. However, unlike the present study, the follow-up period was only 1 year. As expected in multivariate analysis a low-grade of OA was also linked to a higher level of satisfaction.

The present study has some limitations. It is a retrospective analysis of a single-surgeon case series. Another limitation is that we could not group patients into satisfied and dissatisfied as used for traditional PASS value calculation due to the very low number of patients in the dissatisfied group, which was probably due to a low number of large tears also preventing the detection of the effect of tendon healing on outcomes. We believe the method used in the present study to discriminate HS from other groups is more relevant at longer-term follow-ups. Additionally, with a larger number of patients future studies could still find the effect of tendon healing on outcomes at small to medium-sized tears.

In conclusion, patient satisfaction mostly depends on patient-related factors like gender, and smoking rather than tendon healing or degeneration in the medium term after arthroscopic repair of small to medium-sized RC tears.

## Author contributions

**Conceptualization:** Onur Hapa, Emre Acar.

**Supervision:** Onur Hapa, Onur Gürsan.

**Data curation:** Selahaddin Aydemir, Gürhan Tükel.

**Investigation:** Selahaddin Aydemir, Ali Cantürk.

**Resources:** Emre Acar.

**Writing – original draft:** Emre Acar.

**Writing – review & editing:** Emre Acar.

**Methodology:** Berkay Yanik.

**Project administration:** Berkay Yanik.

**Formal analysis:** Ali Balci.
